# The DNA methylation of FOXO3 and TP53 as a blood biomarker of late-onset asthma

**DOI:** 10.1186/s12967-020-02643-y

**Published:** 2020-12-09

**Authors:** Lin Yuan, Leyuan Wang, Xizi Du, Ling Qin, Ming Yang, Kai Zhou, Mengping Wu, Yu Yang, Zhiyuan Zheng, Yang Xiang, Xiangping Qu, Huijun Liu, Xiaoqun Qin, Chi Liu

**Affiliations:** 1 Department of Respiratory Medicine, National Clinical Research Center for Respiratory Diseases, Xiangya Hospital, Central South University, Changsha, Hunan China; 2 Department of Physiology, Xiangya School of Basic Medicine Science, Central South University, Changsha, 410078 Hunan China; 3 Basic and Clinical Research Laboratory of Major Respiratory Diseases, Central South University, Changsha, Hunan China; 4 Centre for Asthma and Respiratory Disease, School of Biomedical Sciences and Pharmacy, Faculty of Health and Medicine, University of Newcastle and Hunter Medical Research Institute, Callaghan, NSW Australia; 5 Research Center of China-Africa Infectious Diseases, Xiangya School of Medicine, Central South University, Changsha, Hunan China

**Keywords:** DNA methylation, Aging, Late-onset asthma, FOXO3, TP53

## Abstract

**Background:**

Late-onset asthma (LOA) is beginning to account for an increasing proportion of asthma patients, which is often underdiagnosed in the elderly. Studies on the possible relations between aging-related genes and LOA contribute to the diagnosis and treatment of LOA. Forkhead Box O3 (FOXO3) and TP53 are two classic aging-related genes. DNA methylation varies greatly with age which may play an important role in the pathogenesis of LOA. We supposed that the differentially methylated sites of FOXO3 and TP53 associated with clinical phenotypes of LOA may be useful biomarkers for the early screening of LOA.

**Methods:**

The mRNA expression and DNA methylation of FOXO3 and TP53 in peripheral blood of 43 LOA patients (15 mild LOA, 15 moderate LOA and 13 severe LOA) and 60 healthy controls (HCs) were determined. The association of methylated sites with age was assessed by Cox regression to control the potential confounders. Then, the correlation between differentially methylated sites (DMSs; *p*-value < 0.05) and clinical lung function in LOA patients was evaluated. Next, candidate DMSs combining with age were evaluated to predict LOA by receiver operating characteristic (ROC) analysis and principal components analysis (PCA). Finally, HDM-stressed asthma model was constructed, and DNA methylation inhibitor 5-Aza-2′-deoxycytidine (5-AZA) were used to determine the regulation of DNA methylation on the expression of FOXO3 and TP53.

**Results:**

Compared with HCs, the mRNA expression and DNA methylation of FOXO3 and TP53 vary significantly in LOA patients. Besides, 8 DMSs from LOA patients were identified. Two of the DMSs, chr6:108882977 (FOXO3) and chr17:7591672 (TP53), were associated with the severity of LOA. The combination of the two DMSs and age could predict LOA with high accuracy (AUC values = 0.924). In HDM-stressed asthma model, DNA demethylation increased the expression of FOXO3 and P53.

**Conclusions:**

The mRNA expression of FOXO3 and TP53 varies significantly in peripheral blood of LOA patients, which may be due to the regulation of DNA methylation. FOXO3 and TP53 methylation is a suitable blood biomarker to predict LOA, which may be useful targets for the risk diagnosis and clinical management of LOA.

## Introduction

Asthma symptoms can occur at any time in life though many patients may develop asthma during childhood. The endotype of asthma has a diversity of distinguished clinical phenotypes which is critical for the diagnosis and treatment of different types of asthma. LOA is defined as a kind of asthma with onset of symptoms in adult life in a patient with no pre-existing, persistent respiratory symptoms [1, 2]. With the worldwide population trend toward enhanced longevity, clinical concerns about LOA are developing which is frequently underdiagnosed in elder age groups. However, the underlying pathogenesis of LOA is still obscure, which have not identified early biomarkers until now. Lung aging has been recognized as an important risk factor and a key pathological basis of LOA [3]. Along with the growth of age, structural and functional changes occur in the lungs of the elderly, leading to an increased risk of pulmonary diseases, more severe pulmonary pathological phenotype and poor clinical treatment [4, 5]. Also, in aging-related chronic obstructive pulmonary disease (COPD) and LOA, aging-related molecular events (such as telomere shortening, epigenetic alterations, ROS accumulation and immunosenescence) are more pronounced [6–8]. The role of lung aging in the pathogenesis of COPD has been extensively studied and confirmed, however, the mechanisms underlying the relations between aging and LOA are still unclear.

FOXO3 and TP53 are two classic genes that regulate aging and longevity [9, 10]. FOXO3 is a member of FOXO transcription factors and belongs to Forkhead family. It is an important transcriptional factor for DNA repair and production of anti-oxidants [11], which is closely related to human longevity [12, 13]. FOXO3 deficiency leads to airspace enlargement, enhanced inflammation and increased sensitivity to COPD after cigarette smoking [14]. Similarly, TP53 has been shown to be involved in the progression of COPD by mediating the senescence of multiple lung cells [15–17]. Although previous studies suggested that the gene polymorphisms in FOXO3 and TP53 are associated with asthma susceptibility [18–20], little is known about the involvement of FOXO3 and TP53 in the pathogenesis of LOA.

Lungs exchange gas with the outside world, which are highly susceptible to external environmental conditions [21]. Therefore, epigenetic modifications have been shown to play an important role in the pathogenesis of asthma. DNA methylation is the most in-depth epigenetic marker in aging-related diseases [22, 23]. It is an important mechanism in the regulation of gene expression which is also closely related to clinical phenotypic changes [24, 25]. As proven, it could be used for risk prediction and early diagnosis in many different diseases [26–28]. Moreover, it is intriguing that aberrant DNA methylation in cancer should be associated with the expression of FOXO3 and TP53, respectively [29, 30]. In the study, we firstly investigated the expression and DNA methylation of FOXO3 and TP53 in peripheral blood of LOA patients. Then, we analyzed the correlation between DMSs and lung function in LOA patients. Finally, we evaluated the feasibility of methylation of FOXO3 and TP53 as blood biomarkers for LOA and determined the regulation of DNA methylation on the expression of FOXO3 and TP53.

## Materials and methods

### Selection of patients with LOA, clinical criteria

Criteria for the diagnosis of asthma were recommended by the 2019 Global Strategy for Asthma Management and Prevention [31]. Briefly, it is defined by the history of respiratory symptoms such as wheeze, shortness of breath, chest tightness and cough that vary over time and in intensity, together with variable expiratory airflow limitation. According to asthma severity, asthma is divided into mild asthma, moderate asthma, and severe asthma. Asthma severity is assessed retrospectively from the level of treatment required to control symptoms and exacerbations. Specific clinical criteria also refer to 2019 Global Strategy for Asthma Management and Prevention [31].

LOA is one of the main endotypes of asthma that have been identified [32]. It refers to people develop asthma symptoms for the first time in adulthood [8], specifically defined as: “asthma with onset of symptoms in adult life in a patient with no pre-existing, persistent respiratory symptoms,” but the definition of age has not been clarified. The cut-off for LOA was set at the age of 13, 40 or 60 years in different researches [1, 2, 33, 34].

### Recruitment of participants and data collection

60 healthy controls (HCs) and 43 previously confirmed LOA patients (> 20 years of age) were selected from the Respiratory Department and the Medical Examination Center of Xiangya Hospital in China from October 2018 to January 2019. After Written informed consent was obtained from each patient, questionnaire information (general condition, smoking history and other respiratory diseases), pulmonary function testing, peripheral blood and induced sputum samples were collected. Lung function test included the spirometric values of FEV_1_, FEV_1_% predicted, FVC, the ratio of FEV_1_ to the FVC, PEF, FEF_75_, FEF_50_ and FEF_25_. The study was approved by No. 20180308 of the Xiangya Hospital Ethics Review Committee and participants provided written consents to participate in this study.

### Sample collection

Peripheral blood and induced sputum samples were collected from the enrolled 60 HCs and 43 LOA patients, respectively. Peripheral blood was collected into 5 ml EDTA anticoagulation tubes and then transferred to a centrifuge tube. After adding 2 volumes of erythrocyte lysate and lysing for 5 min, peripheral blood cells were pelleted by centrifugation.

For sputum induction, each volunteer inhaled a 4.5% saline atomized solution three times for 5 min each time and coughed sputum into a separate cup. Then, four-parts 0.1% dithiothreitol was added to one-part sputum and mixed for 15 min before adding four-parts phosphate-buffered saline. Finally, sputum cells were pelleted by centrifugation and used for RNA extraction after filtering [35].

### RNA extraction, RT-PCR and quantitative RT-PCR

Total mRNA was purified from peripheral blood and induced sputum cells using Trizol (Invitrogen) and quantified by an ultraviolet spectrophotometer (Thermo Fisher Scientific, USA) [36]. 1 µg RNA was reversely transcribed into cDNA using Reverse Transcriptase Kit (Qiagen, Netherlands) in accordance to the manufacturer’s instructions [37]. Then, quantitative RT-PCR was performed using SYBR® Premix Ex Taq™ II system (TaKaRa, Japan) with the CFX96 Touch™ Real-Time PCR Detection System (Bio‐Rad, USA). The PCR conditions were as follows: 95 ℃ for 30 s, 40 cycles of 95 ℃ for 15 s, and 60 ℃ for 30 s. Resulting mRNA levels were normalized to β-actin and expressed as a fold change relative to control samples. The sequences of the primers used are as follows: FOXO3 (forward, CGG ACA AAC GGC TCA CTC T; reverse, GGA CCC GCA TGA ATC GAC TAT), TP53 (forward, AAG TCT GTG ACT TGC ACG TAC TCC; reverse, GTC ATG TGC TGT GAC TGC TTG TAG) and β-actin (forward, TTC CAG CCT TCC TTC CTG GG; reverse, TTG CGC TCA GGA GGA GCA AT).

### DNA extraction, bisulfite treatment, methylation array methods

Genomic DNA was extracted from peripheral blood with the TIANamp Genomic DNA kit (Tiangen Biotech, China) [38]. DNA quality control, bisulfite processing, methylation library construction and high-throughput sequencing were carried out at Genesky Biotechnologies Inc. Shanghai [39]. CpG islands were selected which located between 2 K upstream of the gene transcription start site and 1 K downstream of the first exon, to measure methylation levels. Specific selection methods of CpG islands and detection methods of methylation level are performed according to previous literature [40]. 3 CpG islands of FOXO3 and 2 CpG islands of TP53 were selected. Based on these CpG islands, multiple CpG regions (Targets) from CpG islands in FOXO3 and TP53 were sequenced. The details of the CpG regions are listed in Additional file 1: Table S1. Finally, 171 CpG sites from FOXO3 and 48 CpG sites from TP53 were detected in the methylation assay and the details of the CpG sites can be found in Additional file 2: Table S2. We only retained the raw data with a sequencing quality value Q > 40 (Base sequencing error rate < 0.1%) and reported the percent methylation of every CpG site.

### Animals

This animal study was carried out with the approval No. 2020sydw0178 of Xiangya Animal Protection and Utilization Committee of Central South University. All methods are implemented in accordance with relevant clauses. The mice were placed under the condition of an air‐filtered temperature control unit, where the light and dark cycled alternately for 12 h, with sufficient food and water supply. The HDM‐induced asthma model was constructed according to previous publications [41]. In the last two weeks, 5‐AZA (Sigma‐Aldrich) (1 mg/kg body weight) were injected intraperitoneally 1 h before HDM challenge to inhibit DNA methylation [42]. All mice were killed 48 h after the last challenge.

### Immunohistochemical staining

Lungs were inflated, fixed in 4% paraformaldehyde, and embedded in paraffin blocks and cut into 5 μm sections. Immunohistochemistry staining was performed with the following antibodies: p53 (Ab31333, Abcam), FOXO3 (Ab12162, Abcam). For microscopy, we employed Zeiss Axio Scope.A1 or Zeiss Discovery.V8 Stereo microscopes (Carl Zeiss MicroImaging GmbH, Germany) integrated with an Axio-Cam ICc3 camera (Spectra Service, Ontario, NY). Images were obtained by AxioVision Rel. 4.7 software from Zeiss.

### Statistical analysis

Statistical analyses were conducted using SPSS version 22.0 (IBM Corporation, Armonk, NY, USA). The characteristic data of recruited LOA patients and HCs were presented as Mean ± SD and analyzed with Chi-squared test or Mann–Whitney U test. The mRNA expression and the methylation array of FOXO3 and TP53 were analyzed by One-way ANOVA followed by Dunnett's post hoc test. The method of Benjamin Hochberg was used to control the false discovery rate (FDR). Logistic regression analysis was performed on selected differentially expressed CpG sites, with potential risk factor of age, gender and smoking history [43]. Pearson’s correlation or Spearman’s correlation was used to assess the association between the percentage of methylation of differentially expressed CpG sites and the continuous variables such as FEV_1_, FEV_1_%, FEV_1_/FVC. Receiver operating characteristic (ROC) curves were obtained to elucidate the accuracy of differentially expressed CpG sites in predicting LOA. Age and the methylation percentage of candidate CpG sites were used in principal components analysis (PCA) to identify LOA. A two-tailed p-value < 0.05 was considered statistically significant.

## Results

### Differential expression of FOXO3 and TP53 in peripheral blood and induced sputum of LOA patients and HCs

**Fig. 1:**
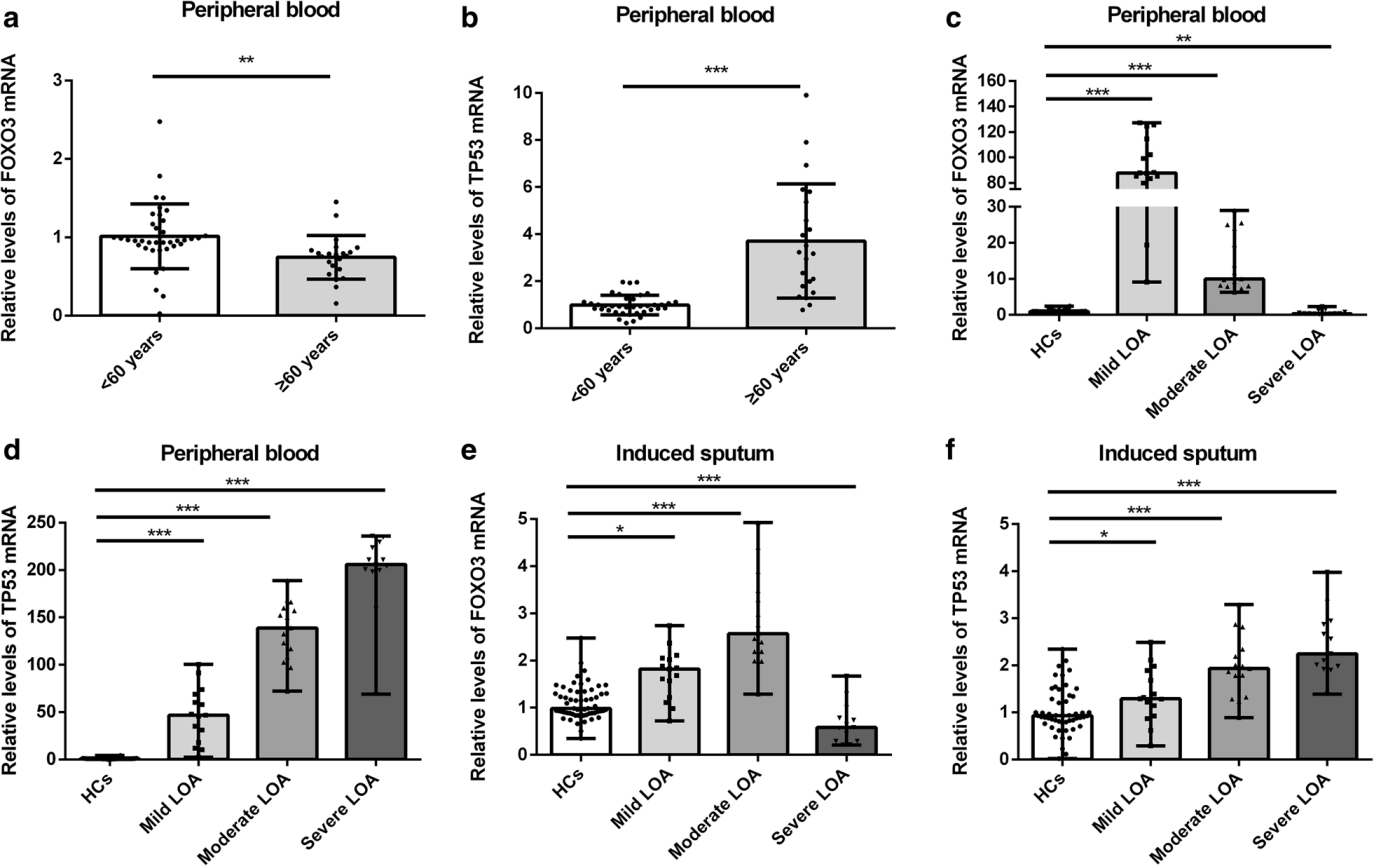
The mRNA level of FOXO3 and TP53 varied significantly in LOA patients. **a**, **b** The mRNA expression of FOXO3 and TP53 in the peripheral blood of HCs < 60 years old and ≥ 60 years old. **c**, **d** The mRNA expression of FOXO3 and TP53 in peripheral blood of LOA patients. **e**, **f** The mRNA expression of FOXO3 and TP53 in induced sputum of LOA patients. *p < 0.05; **p < 0.01; ***p < 0.001

To detect the expression of FOXO3 and TP53 in LOA patients, we recruited 60 HCs and 43 LOA patients (including 15 mild LOA, 15 moderate LOA, and 13 severe LOA). The percentage of eosinophils and neutrophils in LOA patients was significantly higher than that of HCs. There was no statistical difference in age between these groups (Table 1). By comparing the mRNA expression in the peripheral blood of HC < 60 years old and ≥ 60 years old, it was found that the expression of FOXO3 and TP53 was related to age (Fig. 1a, b). The expression change of TP53 and FOXO3 in peripheral blood and induced sputum was consistent. The mRNA expression of FOXO3 increased in mild LOA patients and decreased in severe LOA patients (Fig. 1c, e). While the expression of TP53 decreased both in mild LOA patients and severe LOA patients (Fig. 1d, f). Furthermore, we also performed correlation analyses between the FOXO3 and TP53 mRNA levels and lung function indicators in LOA patients. The result showed that their mRNA expression had varying degrees of correlation with lung function (Additional file 3: Table S3).

**Table 1:** Demographic characteristics of the HCs and LOA patients

	HCs	Mild LOA	Moderate LOA	Severe LOA
Number of subjects	60	15	15	13
Age	53.96 ± 6.89	57.60 ± 13.11	55.60 ± 8.80	50.14 ± 10.25
Female (%)	83.33	73.33	80.00	46.15*
Smoking history (%)	13.33	13.33	6.67	15.38
FEV_1_	2.87 ± 0.21	2.22 ± 0.60*	1.78 ± 0.38*	1.14 ± 0.47*
FEV_1_% predicted	0.94 ± 0.27	0.89 ± 0.57*	0.70 ± 0.06*	0.41 ± 0.14*
FVC	4.07 ± 0.61	3.11 ± 0.80*	2.95 ± 0.85*	2.39 ± 0.78*
FEV_1_/FVC	0.84 ± 0.05	0.71 ± 0.07*	0.62 ± 0.10*	0.48 ± 0.12*
PEF	8.37 ± 0.94	6.22 ± 1.85*	4.26 ± 0.85*	2.83 ± 1.16*
FEF_75_	0.89 ± 0.37	0.72 ± 0.20*	0.45 ± 0.14*	0.21 ± 0.10*
FEF_50_	0.84 ± 0.31	0.49 ± 0.15*	0.33 ± 0.13*	0.13 ± 0.06*
FEF_25_	0.74 ± 0.25	0.37 ± 0.12*	0.23 ± 0.07*	0.15 ± 0.07*
Blood eosinophilia (%)	1.27 ± 3.29	4.03 ± 3.91*	8.22 ± 6.48*	7.53 ± 6.60*
Blood neutrophils (%)	40.76 ± 17.47	58.82 ± 15.91*	60.17 ± 12.29*	71.39 ± 10.44*
Sputum eosinophils (%)	1.11 ± 0.53	3.58 ± 1.80*	9.72 ± 7.24*	11.56 ± 9.35*
Sputum neutrophils (%)	25.05 ± 3.26	31.98 ± 7.35*	33.37 ± 6.31*	42.02 ± 10.29*

Data are presented as Mean ± SD. *p < 0.05, HCs VS LOA patients (Chi-squared test for Female and Smoking history; Unpaired t test for other demographic Characteristics)

### DNA methylation of FOXO3 and TP53 in peripheral blood of patients with LOA and HCs

**Fig. 2:**
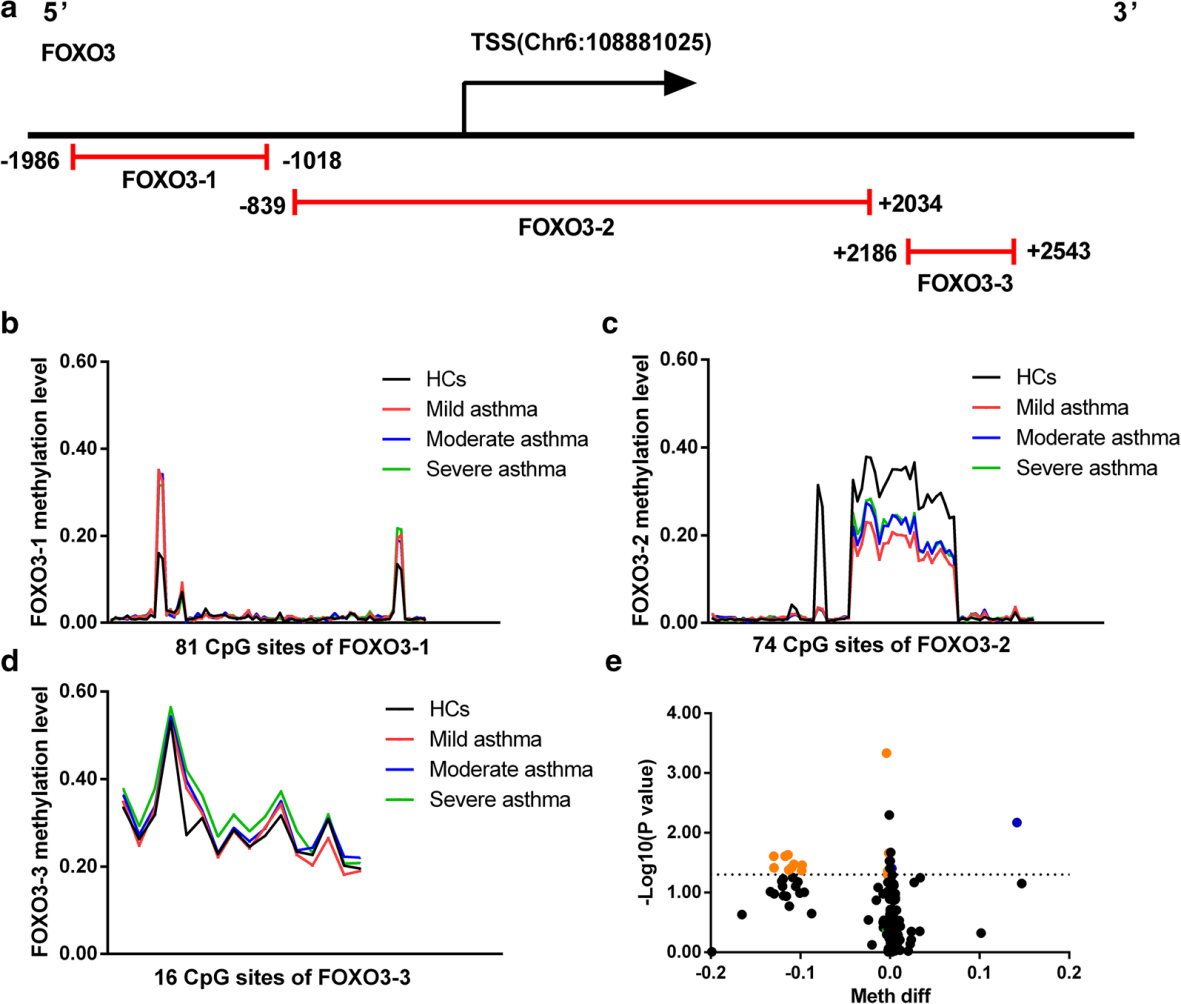
Variable DNA methylation of FOXO3 gene’s CpG islands in LOA. **a** Schematic representation of CpG islands sequenced FOXO3. CpG islands are indicated in Red lines. The Range of each islands is indicated by its relative distance (in bp) to TSS; **b**–**d** Mean DNA methylation level of the three CpG islands (FOXO3-1, FOXO3-2, FOXO3-3) are presented from LOA patients and HCs, respectively; **e** Volcano plot of DMSs in FOXO3 between LOA patients and HCs. The up-regulated sites were presented as blue dots and down-regulated sites were presented as orange dots

**Fig. 3:**
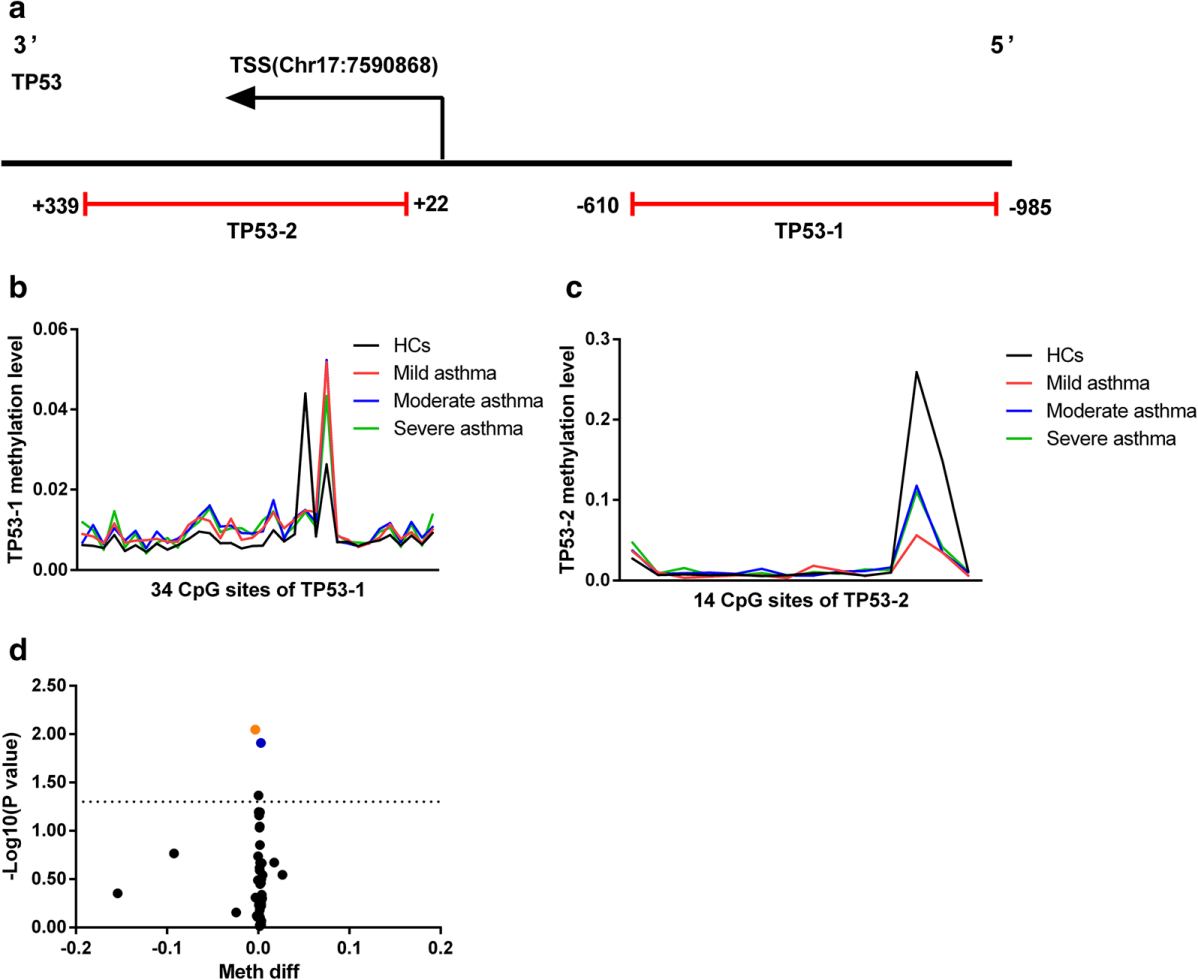
Variable DNA methylation of TP53 gene’s CpG islands in LOA. **a** Schematic representation of CpG islands sequenced TP53; **b**, **c** Mean DNA methylation level of the two CpG islands (TP53-1, TP53-2) from LOA patients and HCs, respectively; **d** Volcano plot of DMSs in TP53 between LOA patients and HCs

Since DNA methylation of CpG sites in the promoter (including the TSS) and first exon regions is closely related to gene transcription [44], we assessed CpG islands located between 2 K upstream of the gene transcription start site and 1 K downstream of the first exon. 3 CpG islands of FOXO3 including 171 CpG sites (Fig. 2a) and 2 CpG islands of TP53 including 48 CpG sites were selected (Fig. 3a). The details of the CpG islands was listed in Table 2. Then, the DNA methylation level of these CpG sites in FOXO3 and TP53 was evaluated. Continuous decreased methylation pattern of CpG sites in specific regions of FOXO3-2 was detected in mild, moderate and severe LOA patients (Fig. 2b–d), while the dominant methylated sites of TP53 was relatively scattered (Fig. 3b, c). The volcano plot also showed that the methylation levels of these CpG sites in FOXO3 (Fig. 2e) and TP53 (Fig. 3d). At *p*-value < 0.05 and the absolute value of meth diff > 0.1%, 15 methylated sites in FOXO3 and 2 methylated sites in TP53 were associated with LOA.

**Table 2:** Details of the CpG islands of FOXO3 and TP53

CpG islands	Chr	mRNA strand	TSS	Start	End	Length	Distance	CpG sites
FOXO3-1	6	+	108881025	108879039	108880007	968	− 1018	81
FOXO3-2	6	+	108881025	108880186	108883059	2873	− 839	74
FOXO3-3	6	+	108881025	108883211	108883568	357	2186	16
TP53-1	17	−	7590868	7591853	7591478	375	− 610	34
TP53-2	17	−	7590868	7590846	7590529	317	22	14

TSS: The mRNA transcription initiation site; Start: The starting position of the CpG island on the reference genome; End: The end position of the CpG island on the reference genome; Length: the CpG island length; Distance: The distance from the CpG island to the TSS

To assess the effect of DMSs more accurately on LOA, logistic regression with age, gender or smoking history was used to determine other possible potential risk factors. The results demonstrated that the age of participants in the study was related to LOA but not to gender and smoking history (data not shown). Hence, a logistic regression analysis with potential risk factors adjustment was further performed. Finally, 7 DMSs in FOXO3 and 1 DMS in TP53 were statistically significant, and the absolute value of methylation difference between the 8 sites ranged from 0.31% to 11.70% (Table 3).

**Table 3:** Differences in methylation sites of FOXO3 and TP53 in LOA patients

CpG Site	CpG island	Mean difference methylation	FDR adjusted *p*-value	*p*-value (logit)	AUC	*p*-value (AUC)
chr6:108879441	FOXO3-1	− 0.39%	< 0.001*	0.013*	0.925	< 0.001*
chr6:108879922	FOXO3-1	− 0.22%	0.043*	0.026*	0.895	< 0.001*
chr6:108880271	FOXO3-2	− 0.08%	0.005*	0.048*	0.887	< 0.001*
chr6:108882982	FOXO3-2	− 11.70%	0.025*	0.041*	0.887	< 0.001*
chr6:108882977	FOXO3-2	− 11.39%	0.023*	0.035*	0.878	< 0.001*
chr6:108882964	FOXO3-2	− 11.00%	0.038*	0.046*	0.880	< 0.001*
chr6:108882825	FOXO3-2	− 10.72%	0.034*	0.049*	0.885	< 0.001*
chr17:7591672	TP53-1	− 0.31%	0.009*	0.043*	0.898	< 0.001*

Differential methylation analysis was conducted between LOA patients and HCs in peripheral blood. The method of Benjamin Hochberg was used to control the false discovery rate (FDR), *p < 0.05; Age factor was adjusted in the logistic regression analysis

### Potential correlation between DMSs and lung function in LOA patients

**Fig. 4:**
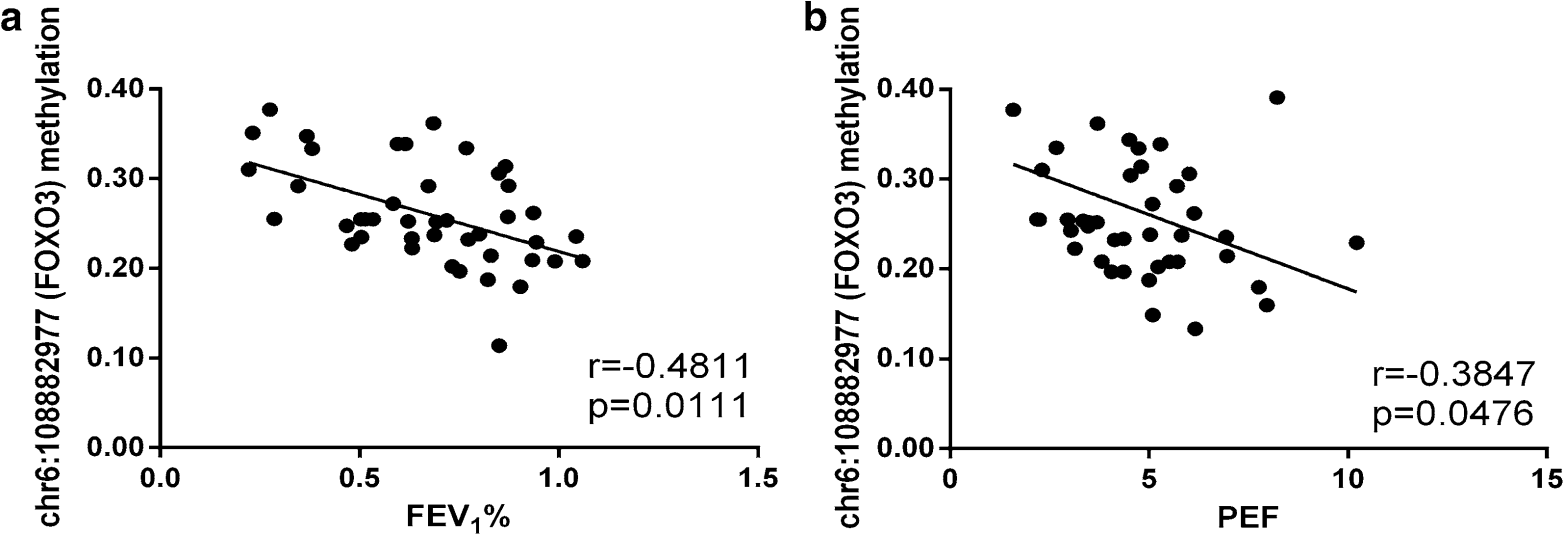
Correlation analysis between chr6:108882977 (FOXO3) and clinical lung function. **a**, **b** The methylation level of chr6:108882977 (FOXO3) was negatively correlated with FEV_1_% and PEF

**Fig. 5:**
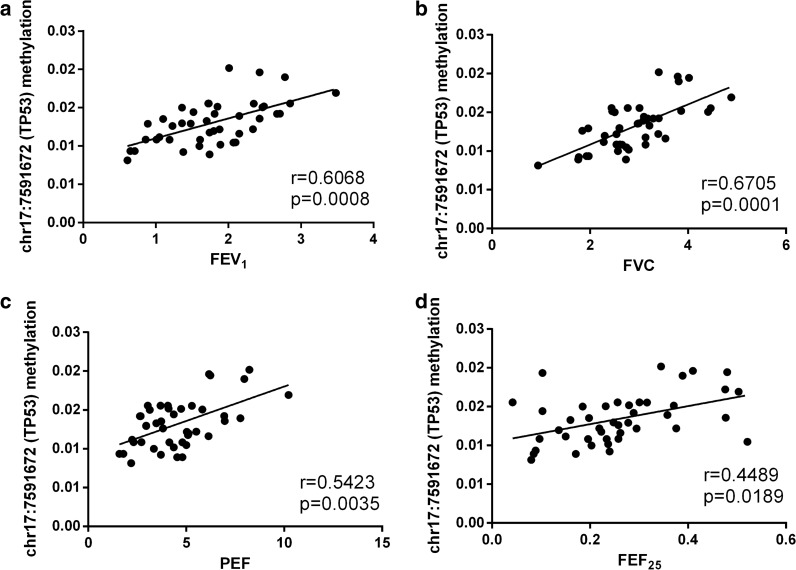
Correlation analysis between chr17:7591672 (TP53) and clinical lung function. **a**–**d** The methylation level of chr17:7591672 (TP53) was positively correlated with FEV_1_, FVC, PEF and FEF_25_

To elucidate that DNA methylation of FOXO3 and TP53 is associated with the severity or exacerbation of LOA, we further analyzed the correlation between the 8 DMSs and lung function indicators of LOA patients, as pulmonary function is an important evaluation basis for the classification and control of asthma severity. Correlation analyses revealed that 2 DMSs were related to changes in lung function indicators (Additional file 4: Table S4). In particular, elevated methylation level of chr6:108882977 (FOXO3) was associated with reduced FEV_1_% and PEF (Fig. 4a, b); methylation level of chr17:7591672 (TP53) was positively related to the changes of FEV_1_, FVC, PEF and FEF_25_ (Fig. 5a–d). Moreover, the methylated site chr6:108882982 (FOXO3) appeared to be associated with decreased lung function, though there was no significant statistical difference (Additional file 4: Table S4).

### DNA methylation level of chr6:108882977 and chr17:591672 as biomarker of LOA

**Fig. 6:**
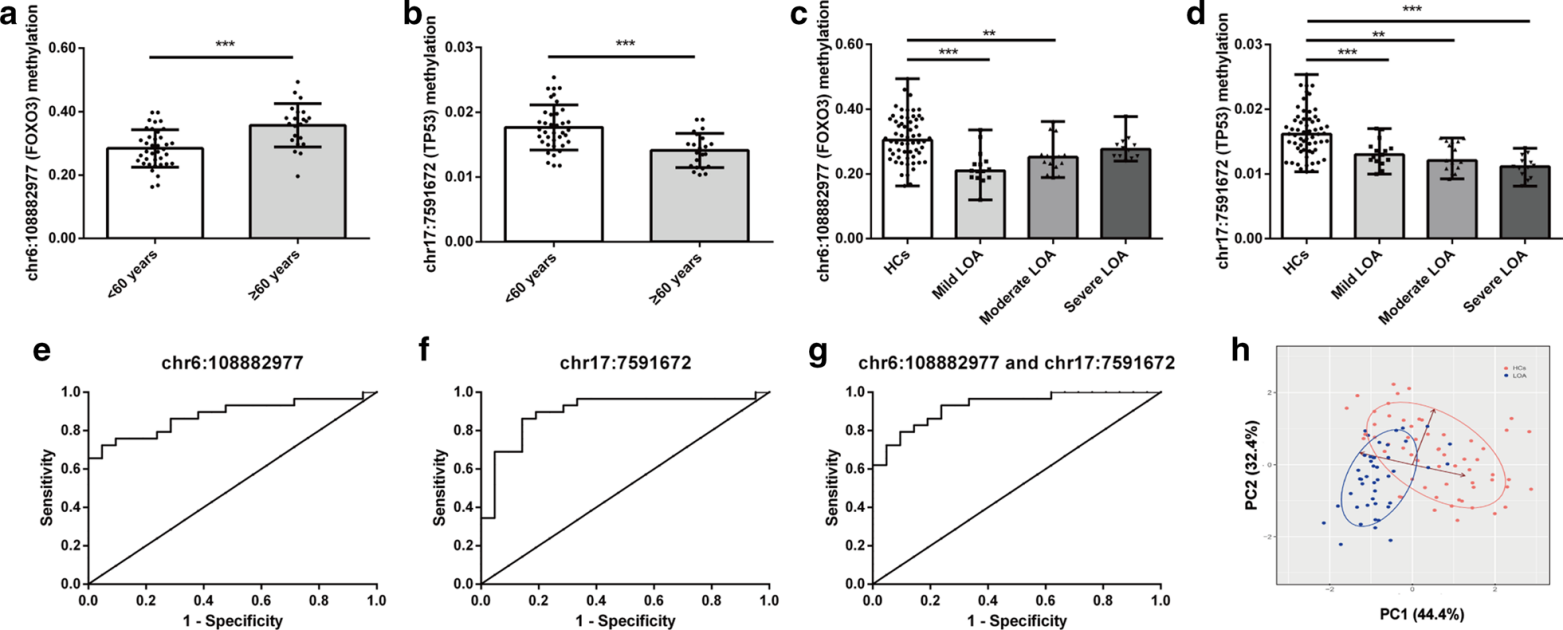
DNA methylation levels of chr6:108882977 (FOXO3) and chr17:7591672 (TP53) and their accuracy in predicting LOA. **a**, **b** The methylation levels of chr6:108882977 (FOXO3) and chr17:7591672 (TP53) in the peripheral blood of HCs < 60 years old and ≥ 60 years old. **c**, **d** The methylation levels of chr6:108882977 (FOXO3) and chr17:7591672 (TP53) in peripheral blood of LOA patients and HCs. **e** ROC curve of DMS (chr6:108882977) and age. **f** ROC curve of DMS (chr17:7591672) and age. **g** ROC curve of the two DMSs and age. (H) A PCA plot consisting of the two DMSs and age. *p < 0.05; **p < 0.01; **p < 0.001

Since the two DMSs (chr6:108882977 and chr17:7591672) were related to age and lung function indicators (Fig. 6a, b), we further analyzed the accuracy of these two DMSs to predict LOA. The methylation levels of chr6:108882977 (FOXO3) decreased in mild and moderate LOA patients (Fig. 6c). While the level of chr17:7591672 (TP53) decreased both in mild LOA patients and severe LOA patients (Fig. 6d). Our previous results demonstrated that age was associated with LOA, so ROC curve analysis was performed after age integration with methylation of chr6:108882977 or chr17:7591672, respectively. ROC curve analysis showed that the chr6:108882977 or chr17:7591672 had high AUC values of 0.878 and 0.898, respectively (Table 3 and Fig. 6e, f). Further, the AUC value increased to 0.924 (Fig. 6g) when ROC analysis was performed in combination with the two DMSs and ages. The PCA plot consisting of the two DMSs and age also demonstrated that the methylation levels of chr6:108882977 (FOXO3) and chr17:7591672 (TP53) could distinguish LOA patients from HCs effectively (Fig. 6h). For the other 6 DMSs, we also performed ROC curve analysis, and the results were shown in Table 3 and Additional file 5: Figure S1.

### DNA demethylation further increased the expression of FOXO3 and P53 in HDM-stressed asthma model

**Fig. 7:**
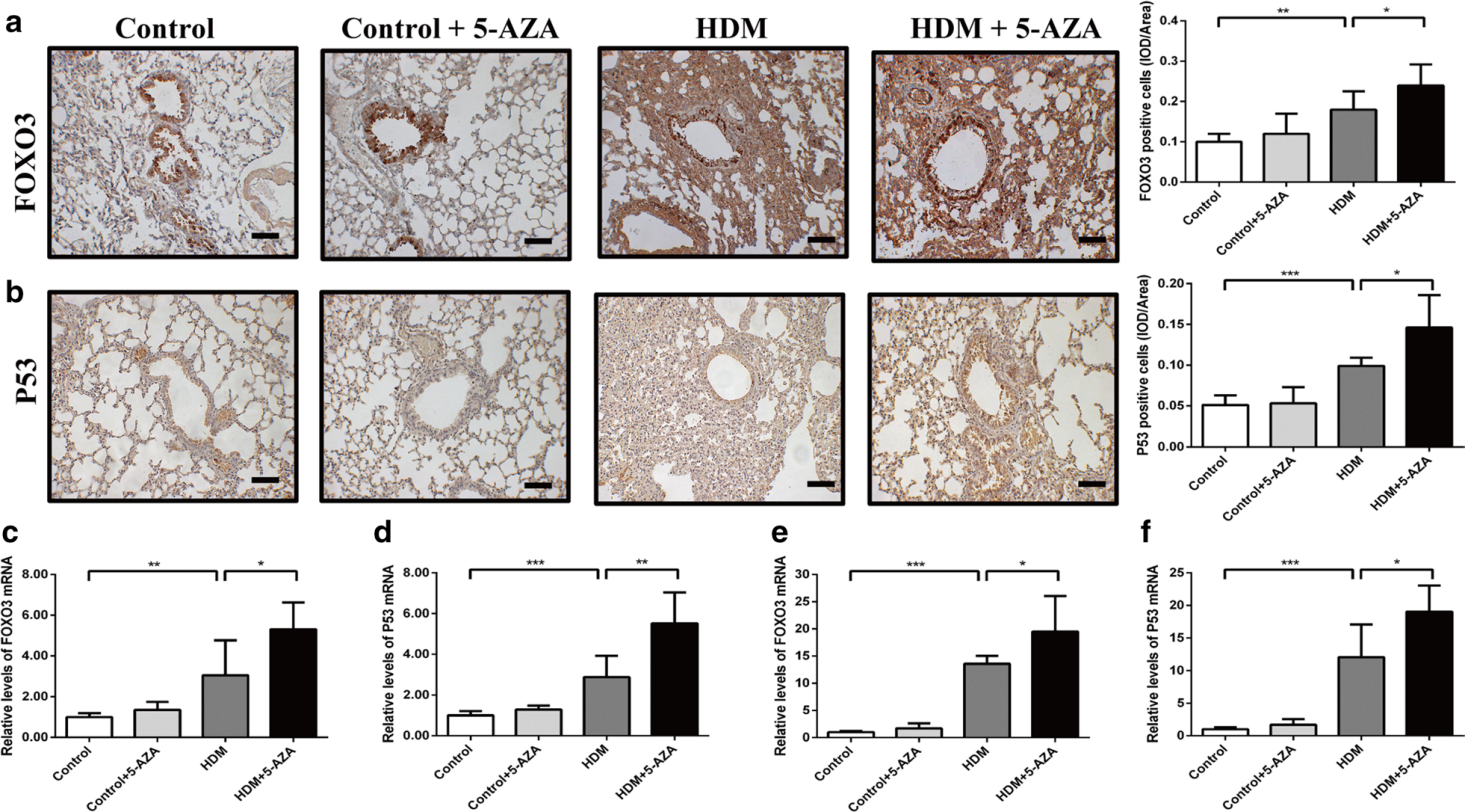
DNA demethylation increased the expression of FOXO3 and P53 in HDM-stressed mice. **a**, **b** The protein expression of FOXO3 and P53 in the lung tissue of asthma mice after 5-AZA treatment, bars: 50 μm. **c**, **d** The mRNA expression of FOXO3 and P53 in the BALF of asthma mice after 5-AZA treatment. **e**, **f** The mRNA expression of FOXO3 and P53 in the peripheral blood of asthma mice after 5-AZA treatment. *p < 0.05; **p < 0.01; ***p < 0.001

To determine the regulation of DNA methylation on the expression of FOXO3 and TP53, 5‐AZA were used to block DNA methylation in HDM‐stressed asthma model. The expression of FOXO3 and P53 in lung tissue, BALF and peripheral blood were increased after HDM stress. And the administration of 5-AZA further increased the expression of FOXO3 and P53 compared to HDM-stressed group (Fig. 7a–f). These results indicated that DNA demethylation was involved in regulating the expression of FOXO3 and TP53 in asthma patients.

## Discussion

Epidemiological studies in recent years have shown that LOA is increasing because of the aging of the population [45]. Moreover, LOA patients are at a higher risk for morbidity and mortality than younger asthma patients [46]. Interestingly, accumulating studies have suggested that the high incidence of LOA is closely related to lung aging [8]. As DNA methylation is the most intensively studied epigenetic mark in aging related studies which represents a very stable sign, aging-related DNA methylation may be used as a valuable biomarker for LOA screening [47, 48]. Here, our study demonstrated that the expression and methylation of two classic aging-related genes (FOXO3 and TP53) vary significantly in LOA patients. Besides, 8 DMSs were identified from LOA patients. Two of the DMSs were associated with the severity of LOA. The combination of the two DMSs and age could predict LOA with high accuracy. Moreover, the regulation of DNA methylation on the expression of FOXO3 and TP53 was determined through in vivo experiments.

FOXO3 is an evolutionarily conserved transcription factor involved in a wide spectrum of biological processes, including aging, apoptosis, and tumor [49]. Previous studies have demonstrated single nucleotide polymorphisms in FOXO3 in peripheral blood of asthma patients [19, 50], and the expression of FOXO3 decreased significantly in the lungs of smokers or COPD patients [14]. It has also found a significant reduction of FOXO3 expression in the bronchial epithelial cells of asthmatic patients [51]. Our results showed that smoking history had nothing to do with LOA. Because the proportion of smokers in our samples was relatively small, we speculated that the altered methylation levels and expression of FOXO3 and TP53 may be caused by other factors like air pollution and occupational exposures [52, 53]. The results further demonstrated that the mRNA expression of FOXO3 was elevated in patients with mild to moderate LOA, whereas decreased in patients with severe LOA. FOXO3 had a pivotal role in inhibiting apoptosis, resisting oxidation, extending cell lifespan and preventing against aging-related diseases [54]. The increased FOXO3 expression may be due to the trigger of protective self-defense mechanisms in mild and moderate LOA patients. TP53 is one of the key genes involved in aging and plays a key role in aging by inhibiting the cell cycle [55, 56]. Studies have verified that genetic polymorphisms in TP53 are associated with asthma susceptibility [18]. It is also shown that prolonged exposure to cigarette smoke extract increases p53 protein expression in the lungs of mice which is consistent with our results [57]. Interestingly, we noticed that the expression changes of FOXO3 and TP53 in the peripheral blood of LOA patients reached ten times or more, which was similar to the expression changes in HDM-stressed mice model. After HDM stress, the expression change of FOXO3 and P53 in peripheral blood was much larger than that in lung tissue and BALF. As DNA methylation is tissue specific [58], there may be unpredictable differences in methylation level between peripheral blood sample and lung tissue sample. Moreover, both FOXO3 and TP53 have been verified to regulate their own expression via different positive feedback loops [59–62]. The regulation of the expression of FOXO3 and TP53 by DNA demethylation may initiate these positive feedback and then amplify expression changes. However, it is still unclear whether the positive feedback loop is based on DNA methylation, transcription or translation, which still requires further research.

In addition, our results also revealed that there were 8 CpG sites associated with LOA in the CpG islands of FOXO3 and TP53. Among them, chr6:108882977 (FOXO3) and chr17:7591672 (TP53) were closely related to the severity of LOA. On this basis, we further explored the possible regulation of gene expression by the two DMSs and the feasibility of the two DMSs as LOA biomarkers. Hypermethylation modifications usually result in decreased target gene expression [63, 64]. Consistent with this, methylation levels of chr6:108882977 (FOXO3) showed a negative regulation on FOXO3 expression. However, it was worth noting that FOXO3 mRNA expression was down-regulated in severe LOA, while there was no significant difference in DNA methylation levels of chr6:108882977. Severe asthma had complex pathological mechanisms and substantial differences in cytokine production compared to mild and moderate asthma, particularly in Th1 and Th17 cells. And the cytokine expression patterns of these cells triggered interactions among pathways [65]. Thus, we speculated that cytokine pathway interactions in severe LOA may affect FOXO3 expression, not just DNA methylation, which still needs further research to verify. Most notably, the ROC curve analysis and PCA suggested that the DNA methylation levels of chr6:108882977 and chr17:7591672 in the peripheral blood can effectively distinguish LOA patients from HCs. This correlation strongly suggests that the methylation level of the chr6:108882977 in FOXO3 and chr17:7591672 in TP53 can be used as a blood biomarker to predict and screen LOA. This DNA methylation detection from peripheral blood is convenient and cost-effective, which is an ideal choice to be the novel biomarkers of LOA.

Although these specific CpG sites may provide blood biomarker to predict and screen LOA, there are still some limitations in this study. The first one is the relatively limited sample size, a new LOA cohort or Long‐standing asthma cohort (Age of onset < 12 years) should be collected in our further work. Besides, the regulation of the specific CpG site methylation on the expression of FOXO3 and TP53 still needs further verification. Moreover it have been verified that FOXO3 and TP53 are expressed in eosinophils and neutrophils [66–69]. Further studies are needed to explore the cellular and molecular mechanisms by which DNA methylation regulated the expression of FOXO3 and TP53.

## Conclusions

In summary, this study verified the altered mRNA expression of FOXO3 and TP53 in the peripheral blood of LOA patients, which may be due to the regulation of DNA methylation. Besides, the DNA methylation levels of the chr6:108882977 in FOXO3 and chr17:7591672 in TP53 in peripheral blood were associated with the clinically phenotypic which may be used as effective blood biomarkers of LOA. These results provide some useful insights into the molecular mechanisms of aging-related genes in LOA which provide some potential useful targets for early prediction and screening of LOA.

## Supplementary Information

**Additional file 1: Table S1.** Details of CpG regions in the CpG islands of FOXO3 and TP53.

**Additional file 2: Table S2.** Methylation level of each CpG site in the CpG islands of FOXO3 and TP53.

**Additional file 3: Table S3.** Correlation between mRNA expression of FOXO3 and TP53 and clinical parameters in LOA patients.

**Additional file 4: Table S4.** Correlation between DNA methylation levels and clinical parameters in LOA patients.

**Additional file 5: Figure S1.** The sensitivity and specificity of 6 DMSs in predicting LOA.

## Data Availability

Not applicable.

## Abbreviations

Forkhead Box O3: FOXO3
DMSs: Differentially methylated sites
ROC: Receiver operating characteristic
PCA: Principal components analysis
5-AZA: 5-Aza-2′-deoxycytidine
COPD: Chronic obstructive pulmonary disease
FEV_1_: Forced expiratory volume in one second
FEV_1_%: Forced expiratory volume in one second as percentage of predicted volume
FVC: Forced vital capacity
PEF: Peak expiratory flow
FEF: Forced expiratory flow
