# Correction to: The DNA methylation of FOXO3 and TP53 as a blood biomarker of late-onset asthma

**DOI:** 10.1186/s12967-021-02777-7

**Published:** 2021-03-16

**Authors:** Lin Yuan, Leyuan Wang, Xizi Du, Ling Qin, Ming Yang, Kai Zhou, Mengping Wu, Yu Yang, Zhiyuan Zheng, Yang Xiang, Xiangping Qu, Huijun Liu, Xiaoqun Qin, Chi Liu

**Affiliations:** 1 Department of Respiratory Medicine, National Clinical Research Center for Respiratory Diseases, Xiangya Hospital, Central South University, Changsha, Hunan China; 2 Department of Physiology, Xiangya School of Basic Medicine Science, Central South University, Changsha, 410078 Hunan China; 3 Basic and Clinical Research Laboratory of Major Respiratory Diseases, Central South University, Changsha, Hunan China; 4 Centre for Asthma and Respiratory Disease, School of Biomedical Sciences and Pharmacy, Faculty of Health and Medicine, University of Newcastle and Hunter Medical Research Institute, Callaghan, NSW Australia; 5 Research Center of China-Africa Infectious Diseases, Xiangya School of Medicine, Central South University, Changsha, Hunan China

## Correction to: J Transl Med (2020) 18:467 https://doi.org/10.1186/s12967-020-02643-y

Following publication of the original article [1], the authors would like to correct the Funding section

The Funding section originally read:

This work was funded by Grants #82070034, #81970033, #31900424, #81570026, #81670002 and #3167188 from the NSFC; Grants #2017JJ2402, #2019JJ50760 from the Hunan Natural Science Foundation; Grant #20K142 from open Foundation of Hunan College Innovation Program and Grants #2018zzts813, #2018zzts812, #2019zzts327, #2019zzts1008 from the Fundamental Research Funds for the Central Universities of Central South University.

The Funding section should read:

This work was funded by Grants #82070034, #81970033, #31900424, #81670002 and #31671188 from the NSFC; grants #2020JJ4776, #2020JJ4688, #2019JJ50760 from the Hunan Natural Science Foundation; grant #20K142 from open Foundation of Hunan College Innovation Program and grants #2019zzts327, #2019zzts1008 from the Fundamental Research Funds for the Central Universities of Central South University.

The original article [1] has been corrected.
