# Supplementary material for: The DNA methylation of FOXO3 and TP53 as a blood biomarker of late-onset asthma
Source: J Transl Med. 2020 Dec 9;18:467. doi: 10.1186/s12967-020-02643-y (PMC7726856; doi:10.1186/s12967-020-02643-y)
Supplement: Supplementary file 1 — Additional file 1: Table S1. Details of CpG regions in the CpG islands of FOXO3 and TP53. [file 12967_2020_2643_MOESM1_ESM.doc]

| **CpG islands** | **Chr** | **mRNA strand** | **Target** | **TSS** | **Start** | **End** | **Length** | **Target strand** | **Distance** | **Primer sequences** |
| --- | --- | --- | --- | --- | --- | --- | --- | --- | --- | --- |
| FOXO3-1 | 6 | + | FOXO3-1#1 | 108881025 | 108879672 | 108879457 | 216 | - | -1568 | F:GTTGATTGGTYGYGGTTATTAGTTTTT  R:CTTCCTCCCRTCCRCTATCC |
|  |  |  | FOXO3-1#2 | 108881025 | 108879573 | 108879347 | 227 | - | -1678 | F:AAGGAAGGAGAGAGAGGAGAGG  R:CRaacccTAACRCCCCCTAC |
|  |  |  | FOXO3-1#3 | 108881025 | 108879202 | 108879039 | 164 | - | -1986 | F:GTTTGTTTATTAGYGTAYGTGGGTGTT  R:AAAAACAACCTAACTATCTCRCRAACAAC |
|  |  |  | FOXO3-1#4 | 108881025 | 108879622 | 108879861 | 240 | + | -1403 | F:ggYGgAGAGGGGYGTTTG  R:CCCAACCRCCTCCRAACT |
|  |  |  | FOXO3-1#5 | 108881025 | 108879746 | 108880007 | 262 | + | -1279 | F:TGGATAYGATGATATTATTTGTTTGTAYG  R:ACACCAAAAATCTACACACTAATTCataaa |
| FOXO3-2 | 6 | + | FOXO3-2#1 | 108881025 | 108880713 | 108880450 | 264 | - | -575 | F:TTYGggagaggagggagaag  R:ACRACCAACATCTTATCTCRAATATTACC |
|  |  |  | FOXO3-2#2 | 108881025 | 108880565 | 108880357 | 209 | - | -668 | F:GGTATTAGGGGGTTTTTGGTTTG  R:ATCRAATCTATAAACATCAACTTATCRAAAT |
|  |  |  | FOXO3-2#3 | 108881025 | 108880445 | 108880186 | 260 | - | -839 | F:TGGGTAGGGTTATYGYGGTGT  R:CCTTCTCAATTATTCRAAATCTACTCTT |
|  |  |  | FOXO3-2#4 | 108881025 | 108883059 | 108882787 | 273 | - | 1762 | F:TGTTGTTAGTTYGGGGTGGGTA  R:CTACTACAACCTCAACAACCRCTACCA |
|  |  |  | FOXO3-2#5 | 108881025 | 108881637 | 108881428 | 210 | - | 403 | F:AGAAAGAAGGGGGAGTAGTAGTTTAG  R:AATTTTCTAACAACCCRAAAAACTTAAC |
| FOXO3-3 | 6 | + | FOXO3-3#1 | 108881025 | 108883211 | 108883464 | 254 | + | 2186 | F:GGTTTYGGAGGGGTGAATG  R:CCAACCCCTCCTCCAAAC |
|  |  |  | FOXO3-3#2 | 108881025 | 108883301 | 108883568 | 268 | + | 2276 | F:AYGGATAGGAGTATATTTGTTGGATTTT  R:TACCTATAAATATTAACACCCACAAACTAAA |
| TP53-1 | 17 | - | TP53-1#1 | 7590868 | 7591853 | 7591611 | 243 | - | -985 | F:AGGTAGAAGATTTTYGGGAGGAG  R:ACAACCACRAAAAACCCTAAAAC |
|  |  |  | TP53-1#2 | 7590868 | 7591671 | 7591478 | 194 | - | -803 | F:GGGAYGTGAAAGGTTAGAAGGT  R:AAATACAAAACCTACTACRCCCTCTACAA |
| TP53-2 | 17 | - | TP53-2#1 | 7590868 | 7590846 | 7590610 | 237 | - | 22 | F:TTTATGTGTTTAAGATTGGYGTTAAAAGT  R:CCCAACRATTTTCCCRAACTAAA |
|  |  |  | TP53-2#2 | 7590868 | 7590796 | 7590529 | 268 | - | 72 | F:GAGTTATYGTTTAGGGAGTAGGTAGTTGT  R:ACTCCTATAATAATCCCTCTAACCAAAC |

**Table S1: Details of CpG regions in the CpG islands of FOXO3 and TP53.**

Start: The starting position of the product on the reference genome; End: The end position of the product on the reference genome; Length: the product length; Target strand: The product orientation; Distance: The distance from the product to the TSS.
