# Supplementary material for: The DNA methylation of FOXO3 and TP53 as a blood biomarker of late-onset asthma
Source: J Transl Med. 2020 Dec 9;18:467. doi: 10.1186/s12967-020-02643-y (PMC7726856; doi:10.1186/s12967-020-02643-y)
Supplement: Supplementary file 2 — Additional file 2: Table S2. Methylation level of each CpG site in the CpG islands of FOXO3 and TP53. [file 12967_2020_2643_MOESM2_ESM.doc]

**Table S2: Methylation level of each CpG site in the CpG islands of FOXO3 and TP53.**

| **Target** | **CpG Site** | **Mean**  **(HCs)** | **Mean**  **(LOA)** | **Mean**  **Difference Methylation** | **FDR**  **Adjusted *p*-value** |
| --- | --- | --- | --- | --- | --- |
| FOXO3-1#1 | Chr6:108879635 | 0.005 | 0.006 | 0.002 | 0.706 |
|  | Chr6:108879625 | 0.012 | 0.011 | -0.002 | 0.111 |
|  | Chr6:108879617 | 0.009 | 0.009 | <0.001 | 0.193 |
|  | Chr6:108879611 | 0.010 | 0.009 | <0.001 | 0.464 |
|  | Chr6:108879586 | 0.016 | 0.011 | -0.005 | 0.103 |
|  | Chr6:108879536 | 0.008 | 0.010 | 0.002 | 0.188 |
|  | Chr6:108879516 | 0.007 | 0.013 | 0.006 | 0.945 |
|  | Chr6:108879514 | 0.009 | 0.012 | 0.003 | 0.568 |
|  | Chr6:108879512 | 0.008 | 0.013 | 0.005 | 0.883 |
|  | Chr6:108879506 | 0.008 | 0.013 | 0.006 | 0.940 |
|  | Chr6:108879483 | 0.018 | 0.026 | 0.009 | 0.500 |
|  | Chr6:108879481 | 0.013 | 0.017 | 0.004 | 0.072 |
| FOXO3-1#2 | Chr6:108879536 | 0.008 | 0.011 | 0.003 | 0.154 |
|  | Chr6:108879516 | 0.008 | 0.015 | 0.008 | 0.431 |
|  | Chr6:108879514 | 0.009 | 0.012 | 0.003 | 0.368 |
|  | Chr6:108879512 | 0.007 | 0.015 | 0.009 | 0.692 |
|  | Chr6:108879506 | 0.007 | 0.013 | 0.006 | 0.837 |
|  | Chr6:108879483 | 0.054 | 0.039 | -0.015 | 0.913 |
|  | Chr6:108879481 | 0.052 | 0.030 | -0.022 | 0.423 |
|  | Chr6:108879470 | 0.160 | 0.301 | 0.142 | 0.007 |
|  | Chr6:108879466 | 0.147 | 0.295 | 0.147 | 0.070 |
|  | Chr6:108879451 | 0.029 | 0.021 | -0.008 | 0.105 |
|  | Chr6:108879441 | 0.026 | 0.024 | -0.004 | <0.001 |
|  | Chr6:108879426 | 0.023 | 0.018 | -0.004 | 0.493 |
|  | Chr6:108879415 | 0.035 | 0.028 | -0.008 | 0.316 |
|  | Chr6:108879412 | 0.071 | 0.079 | 0.006 | 0.674 |
|  | Chr6:108879393 | 0.007 | 0.008 | 0.003 | 0.237 |
|  | Chr6:108879387 | 0.008 | 0.009 | 0.001 | 0.435 |
|  | Chr6:108879379 | 0.009 | 0.009 | -0.001 | 0.022 |
|  | Chr6:108879377 | 0.013 | 0.014 | <0.001 | 0.300 |
|  | Chr6:108879370 | 0.014 | 0.015 | -0.001 | 0.106 |
| FOXO3-1#3 | Chr6:108879156 | 0.032 | 0.022 | -0.007 | 0.383 |
|  | Chr6:108879147 | 0.018 | 0.014 | -0.002 | 0.983 |
|  | Chr6:108879141 | 0.015 | 0.014 | -0.001 | 0.030 |
|  | Chr6:108879134 | 0.016 | 0.011 | -0.003 | 0.050 |
|  | Chr6:108879130 | 0.018 | 0.014 | -0.002 | 0.067 |
|  | Chr6:108879128 | 0.018 | 0.020 | 0.001 | 0.645 |
|  | Chr6:108879120 | 0.012 | 0.016 | 0.003 | 0.372 |
|  | Chr6:108879116 | 0.011 | 0.014 | 0.003 | 0.508 |
|  | Chr6:108879114 | 0.010 | 0.010 | 0.002 | 0.065 |
|  | Chr6:108879108 | 0.010 | 0.010 | <0.001 | 0.917 |
|  | Chr6:108879100 | 0.011 | 0.015 | 0.003 | 0.770 |
|  | Chr6:108879098 | 0.016 | 0.022 | 0.003 | 0.884 |
|  | Chr6:108879095 | 0.008 | 0.008 | 0.001 | 0.021 |
|  | Chr6:108879088 | 0.014 | 0.018 | 0.003 | 0.958 |
|  | Chr6:108879086 | 0.006 | 0.009 | 0.002 | 0.040 |
|  | Chr6:108879083 | 0.008 | 0.012 | 0.003 | 0.572 |
|  | Chr6:108879081 | 0.006 | 0.008 | 0.002 | 0.107 |
|  | Chr6:108879072 | 0.006 | 0.008 | 0.001 | 0.083 |
|  | Chr6:108879068 | 0.007 | 0.009 | 0.002 | 0.188 |
| FOXO3-1#4 | Chr6:108879659 | 0.023 | 0.015 | -0.007 | 0.292 |
|  | Chr6:108879661 | 0.003 | 0.007 | 0.005 | 0.069 |
|  | Chr6:108879672 | 0.005 | 0.010 | 0.004 | 0.529 |
|  | Chr6:108879674 | 0.009 | 0.010 | 0.003 | 0.254 |
|  | Chr6:108879676 | 0.006 | 0.009 | 0.002 | 0.118 |
|  | Chr6:108879679 | 0.005 | 0.006 | <0.001 | 0.358 |
|  | Chr6:108879689 | 0.003 | 0.006 | 0.003 | 0.857 |
|  | Chr6:108879697 | 0.007 | 0.007 | 0.002 | 0.077 |
|  | Chr6:108879700 | 0.009 | 0.011 | <0.001 | 0.929 |
|  | Chr6:108879705 | 0.004 | 0.007 | 0.003 | 0.936 |
|  | Chr6:108879712 | 0.005 | 0.006 | <0.001 | 0.506 |
|  | Chr6:108879715 | 0.009 | 0.010 | 0.002 | 0.605 |
|  | Chr6:108879717 | 0.010 | 0.009 | -0.002 | 0.921 |
|  | Chr6:108879720 | 0.012 | 0.009 | -0.002 | 0.867 |
|  | Chr6:108879730 | 0.009 | 0.017 | 0.005 | 0.589 |
|  | Chr6:108879738 | 0.010 | 0.011 | 0.001 | 0.875 |
|  | Chr6:108879740 | 0.013 | 0.012 | -0.002 | 0.095 |
|  | Chr6:108879742 | 0.009 | 0.010 | 0.001 | 0.541 |
|  | Chr6:108879752 | 0.022 | 0.032 | 0.006 | 0.761 |
|  | Chr6:108879773 | 0.019 | 0.027 | 0.005 | 0.922 |
|  | Chr6:108879775 | 0.008 | 0.010 | 0.002 | 0.264 |
|  | Chr6:108879778 | 0.009 | 0.011 | 0.003 | 00.874 |
|  | Chr6:108879799 | 0.006 | 0.008 | 0.002 | 0.811 |
|  | Chr6:108879805 | 0.014 | 0.021 | 0.008 | 0.768 |
|  | Chr6:108879807 | 0.006 | 0.011 | 0.006 | 0.104 |
|  | Chr6:108879809 | 0.007 | 0.011 | 0.004 | 0.484 |
|  | Chr6:108879823 | 0.007 | 0.009 | 0.004 | 0.076 |
|  | Chr6:108879827 | 0.007 | 0.013 | 0.006 | 0.838 |
|  | Chr6:108879832 | 0.008 | 0.012 | 0.003 | 0.464 |
|  | Chr6:108879841 | 0.025 | 0.028 | 0.003 | 0.740 |
| FOXO3-1#5 | Chr6:108879775 | 0.007 | 0.010 | 0.002 | 0.535 |
|  | Chr6:108879778 | 0.008 | 0.010 | 0.004 | 0.822 |
|  | Chr6:108879799 | 0.006 | 0.008 | <0.001 | 0.515 |
|  | Chr6:108879805 | 0.012 | 0.020 | 0.008 | 0.822 |
|  | Chr6:108879807 | 0.008 | 0.010 | 0.004 | 0.494 |
|  | Chr6:108879809 | 0.009 | 0.009 | <0.001 | 0.571 |
|  | Chr6:108879823 | 0.009 | 0.011 | 0.003 | 0.358 |
|  | Chr6:108879827 | 0.007 | 0.011 | 0.003 | 0.195 |
|  | Chr6:108879832 | 0.009 | 0.010 | 0.002 | 0.077 |
|  | Chr6:108879841 | 0.006 | 0.013 | 0.005 | 0.405 |
|  | Chr6:108879848 | 0.135 | 0.172 | 0.027 | 0.068 |
|  | Chr6:108879854 | 0.122 | 0.168 | 0.034 | 0.056 |
|  | Chr6:108879862 | 0.010 | 0.006 | -0.004 | 0.438 |
|  | Chr6:108879876 | 0.007 | 0.008 | <0.001 | 0.128 |
|  | Chr6:108879880 | 0.008 | 0.007 | -0.001 | 0.552 |
|  | Chr6:108879910 | 0.009 | 0.007 | -0.002 | 0.478 |
|  | Chr6:108879922 | 0.011 | 0.009 | -0.002 | 0.043 |
|  | Chr6:108879946 | 0.007 | 0.008 | 0.001 | 0.392 |
| FOXO3-2#1 | Chr6:108880639 | 0.010 | 0.016 | 0.005 | 0.709 |
|  | Chr6:108880630 | 0.005 | 0.009 | 0.005 | 0.945 |
|  | Chr6:108880619 | 0.008 | 0.009 | 0.001 | 0.796 |
|  | Chr6:108880616 | 0.007 | 0.008 | 0.002 | 0.205 |
|  | Chr6:108880604 | 0.009 | 0.010 | 0.001 | 0.881 |
|  | Chr6:108880599 | 0.007 | 0.009 | 0.002 | 0.631 |
|  | Chr6:108880596 | 0.008 | 0.007 | -0.002 | 0.383 |
|  | Chr6:108880579 | 0.007 | 0.009 | 0.003 | 0.234 |
|  | Chr6:108880570 | 0.007 | 0.007 | -0.001 | 0.868 |
|  | Chr6:108880542 | 0.007 | 0.009 | 0.003 | 0.865 |
|  | Chr6:108880540 | 0.006 | 0.006 | <0.001 | 0.864 |
|  | Chr6:108880536 | 0.009 | 0.010 | 0.001 | 0.658 |
|  | Chr6:108880532 | 0.008 | 0.010 | 0.002 | 0.539 |
|  | Chr6:108880529 | 0.007 | 0.009 | 0.002 | 0.984 |
|  | Chr6:108880511 | 0.007 | 0.010 | 0.004 | 0.670 |
|  | Chr6:108880504 | 0.008 | 0.009 | 0.001 | 0.385 |
|  | Chr6:108880488 | 0.007 | 0.010 | 0.003 | 0.992 |
|  | Chr6:108880486 | 0.009 | 0.011 | 0.002 | 0.116 |
| FOXO3-2#2 | Chr6:108880542 | 0.006 | 0.009 | 0.003 | 0.073 |
|  | Chr6:108880540 | 0.006 | 0.007 | <0.001 | 0.430 |
|  | Chr6:108880536 | 0.010 | 0.012 | 0.002 | 0.104 |
|  | Chr6:108880532 | 0.009 | 0.012 | 0.003 | 0.733 |
|  | Chr6:108880529 | 0.006 | 0.010 | 0.004 | 0.447 |
|  | Chr6:108880511 | 0.007 | 0.009 | 0.003 | 0.928 |
|  | Chr6:108880504 | 0.008 | 0.008 | 0.001 | 0.198 |
|  | Chr6:108880488 | 0.007 | 0.008 | 0.001 | 0.058 |
|  | Chr6:108880486 | 0.008 | 0.010 | 0.002 | 0.101 |
|  | Chr6:108880469 | 0.041 | 0.019 | -0.024 | 0.286 |
|  | Chr6:108880452 | 0.032 | 0.020 | -0.013 | 0.082 |
|  | Chr6:108880432 | 0.008 | 0.013 | 0.005 | 0.435 |
|  | Chr6:108880430 | 0.007 | 0.010 | 0.003 | 0.342 |
|  | Chr6:108880412 | 0.006 | 0.007 | 0.001 | 0.687 |
|  | Chr6:108880409 | 0.006 | 0.008 | 0.003 | 0.602 |
| FOXO3-2#3 | Chr6:108880412 | 0.006 | 0.007 | 0.001 | 0.265 |
|  | Chr6:108880409 | 0.006 | 0.008 | 0.001 | 0.177 |
|  | Chr6:108880383 | 0.314 | 0.079 | -0.199 | 0.981 |
|  | Chr6:108880360 | 0.264 | 0.071 | -0.165 | 0.234 |
|  | Chr6:108880321 | 0.004 | 0.007 | 0.003 | 0.814 |
|  | Chr6:108880317 | 0.005 | 0.005 | 0.001 | 0.762 |
|  | Chr6:108880303 | 0.007 | 0.008 | <0.001 | 0.234 |
|  | Chr6:108880271 | 0.009 | 0.009 | -0.001 | 0.005 |
|  | Chr6:108880248 | 0.009 | 0.013 | 0.003 | 0.886 |
|  | Chr6:108880225 | 0.013 | 0.011 | <0.001 | 0.122 |
| FOXO3-2#4 | Chr6:108883037 | 0.325 | 0.229 | -0.109 | 0.056 |
|  | Chr6:108883024 | 0.277 | 0.188 | -0.099 | 0.043 |
|  | Chr6:108883006 | 0.306 | 0.214 | -0.105 | 0.079 |
|  | Chr6:108882982 | 0.378 | 0.281 | -0.117 | 0.025 |
|  | Chr6:108882977 | 0.376 | 0.279 | -0.114 | 0.023 |
|  | Chr6:108882964 | 0.343 | 0.251 | -0.110 | 0.038 |
|  | Chr6:108882941 | 0.287 | 0.191 | -0.112 | 0.041 |
|  | Chr6:108882933 | 0.309 | 0.230 | -0.096 | 0.099 |
|  | Chr6:108882922 | 0.328 | 0.229 | -0.120 | 0.079 |
|  | Chr6:108882916 | 0.350 | 0.250 | -0.119 | 0.112 |
|  | Chr6:108882914 | 0.349 | 0.245 | -0.119 | 0.059 |
|  | Chr6:108882900 | 0.348 | 0.238 | -0.130 | 0.025 |
|  | Chr6:108882898 | 0.355 | 0.246 | -0.129 | 0.038 |
|  | Chr6:108882877 | 0.328 | 0.219 | -0.129 | 0.105 |
|  | Chr6:108882872 | 0.365 | 0.254 | -0.133 | 0.096 |
|  | Chr6:108882869 | 0.259 | 0.178 | -0.100 | 0.102 |
|  | Chr6:108882867 | 0.264 | 0.175 | -0.103 | 0.066 |
|  | Chr6:108882864 | 0.292 | 0.197 | -0.116 | 0.116 |
|  | Chr6:108882852 | 0.274 | 0.165 | -0.121 | 0.065 |
|  | Chr6:108882844 | 0.288 | 0.191 | -0.112 | 0.170 |
|  | Chr6:108882831 | 0.296 | 0.194 | -0.113 | 0.169 |
|  | Chr6:108882825 | 0.270 | 0.174 | -0.107 | 0.034 |
|  | Chr6:108882819 | 0.239 | 0.163 | -0.087 | 0.225 |
|  | Chr6:108882816 | 0.241 | 0.151 | -0.098 | 0.035 |
| FOXO3-2#5 | Chr6:108881611 | 0.005 | 0.007 | 0.001 | 0.104 |
|  | Chr6:108881603 | 0.011 | 0.012 | -0.001 | 0.585 |
|  | Chr6:108881593 | 0.007 | 0.007 | <0.001 | 0.039 |
|  | Chr6:108881587 | 0.012 | 0.019 | 0.007 | 0.197 |
|  | Chr6:108881584 | 0.009 | 0.018 | 0.009 | 0.553 |
|  | Chr6:108881578 | 0.007 | 0.010 | 0.004 | 0.854 |
|  | Chr6:108881569 | 0.013 | 0.024 | 0.011 | 0.919 |
|  | Chr6:108881544 | 0.010 | 0.009 | -0.001 | 0.323 |
|  | Chr6:108881542 | 0.008 | 0.007 | <0.001 | 0.030 |
|  | Chr6:108881538 | 0.010 | 0.010 | <0.001 | 0.202 |
|  | Chr6:108881535 | 0.006 | 0.011 | 0.004 | 0.126 |
|  | Chr6:108881526 | 0.010 | 0.011 | 0.003 | 0.965 |
|  | Chr6:108881511 | 0.006 | 0.009 | 0.002 | 0.912 |
|  | Chr6:108881504 | 0.024 | 0.032 | 0.010 | 0.293 |
|  | Chr6:108881492 | 0.007 | 0.008 | 0.001 | 0.894 |
|  | Chr6:108881487 | 0.005 | 0.006 | 0.002 | 0.367 |
|  | Chr6:108881463 | 0.007 | 0.008 | 0.002 | 0.257 |
|  | Chr6:108881456 | 0.007 | 0.008 | 0.003 | 0.051 |
| FOXO3-3#1 | Chr6:108883281 | 0.334 | 0.362 | 0.024 | 0.617 |
|  | Chr6:108883284 | 0.262 | 0.275 | 0.009 | 0.523 |
|  | Chr6:108883287 | 0.318 | 0.350 | 0.022 | 0.725 |
|  | Chr6:108883298 | 0.532 | 0.549 | 0.011 | 0.618 |
|  | Chr6:108883302 | 0.272 | 0.361 | 0.102 | 0.476 |
|  | Chr6:108883330 | 0.311 | 0.342 | 0.020 | 0.953 |
|  | Chr6:108883340 | 0.228 | 0.246 | 0.008 | 0.705 |
|  | Chr6:108883354 | 0.283 | 0.303 | 0.011 | 0.368 |
|  | Chr6:108883381 | 0.245 | 0.266 | 0.007 | 0.756 |
|  | Chr6:108883399 | 0.270 | 0.302 | 0.024 | 0.450 |
|  | Chr6:108883403 | 0.317 | 0.354 | 0.033 | 0.444 |
|  | Chr6:108883441 | 0.234 | 0.253 | 0.006 | 0.720 |
| FOXO3-3#2 | Chr6:108883330 | 0.315 | 0.341 | 0.013 | 0.918 |
|  | Chr6:108883340 | 0.229 | 0.245 | 0.006 | 0.590 |
|  | Chr6:108883354 | 0.284 | 0.302 | 0.008 | 0.444 |
|  | Chr6:108883381 | 0.246 | 0.264 | 0.003 | 0.607 |
|  | Chr6:108883399 | 0.270 | 0.301 | 0.020 | 0.435 |
|  | Chr6:108883403 | 0.317 | 0.352 | 0.029 | 0.492 |
|  | Chr6:108883441 | 0.236 | 0.252 | 0.001 | 0.571 |
|  | Chr6:108883485 | 0.226 | 0.241 | -0.015 | 0.135 |
|  | Chr6:108883500 | 0.308 | 0.313 | -0.020 | 0.752 |
|  | Chr6:108883503 | 0.202 | 0.220 | -0.007 | 0.397 |
|  | Chr6:108883527 | 0.195 | 0.220 | -0.004 | 0.505 |
| TP53-1#1 | Chr17:7591827 | 0.006 | 0.009 | 0.002 | 0.786 |
|  | Chr17:7591820 | 0.006 | 0.010 | 0.003 | 0.856 |
|  | Chr17:7591816 | 0.006 | 0.006 | 0.001 | 0.064 |
|  | Chr17:7591780 | 0.009 | 0.012 | 0.002 | 0.214 |
|  | Chr17:7591778 | 0.005 | 0.007 | 0.002 | 0.090 |
|  | Chr17:7591773 | 0.006 | 0.008 | 0.002 | 0.661 |
|  | Chr17:7591768 | 0.005 | 0.006 | 0.002 | 0.968 |
|  | Chr17:7591753 | 0.007 | 0.009 | 0.002 | 0.614 |
|  | Chr17:7591751 | 0.005 | 0.007 | 0.002 | 0.578 |
|  | Chr17:7591743 | 0.006 | 0.007 | <0.001 | 0.183 |
|  | Chr17:7591731 | 0.008 | 0.010 | 0.002 | 0.064 |
|  | Chr17:7591722 | 0.010 | 0.012 | 0.002 | 0.140 |
|  | Chr17:7591719 | 0.009 | 0.014 | 0.004 | 0.501 |
|  | Chr17:7591712 | 0.007 | 0.009 | 0.002 | 0.953 |
|  | Chr17:7591700 | 0.007 | 0.010 | 0.003 | 0.598 |
|  | Chr17:7591698 | 0.005 | 0.008 | 0.001 | 0.562 |
|  | Chr17:7591694 | 0.006 | 0.008 | 0.002 | 0.356 |
|  | Chr17:7591687 | 0.006 | 0.010 | 0.004 | 0.216 |
|  | Chr17:7591680 | 0.010 | 0.015 | 0.005 | 0.287 |
|  | Chr17:7591678 | 0.007 | 0.009 | 0.001 | 0.069 |
|  | Chr17:7591672 | 0.009 | 0.006 | -0.003 | 0.009 |
|  | Chr17:7591667 | 0.044 | 0.017 | -0.024 | 0.699 |
| TP53-1#2 | Chr17:7591645 | 0.009 | 0.010 | 0.001 | 0.068 |
|  | Chr17:7591619 | 0.026 | 0.054 | 0.027 | 0.286 |
|  | Chr17:7591592 | 0.007 | 0.008 | 0.001 | 0.242 |
|  | Chr17:7591578 | 0.007 | 0.007 | <0.001 | 0.325 |
|  | Chr17:7591571 | 0.006 | 0.007 | 0.001 | 0.093 |
|  | Chr17:7591565 | 0.007 | 0.007 | <0.001 | 0.043 |
|  | Chr17:7591553 | 0.007 | 0.009 | 0.002 | 0.943 |
|  | Chr17:7591546 | 0.009 | 0.010 | 0.002 | 0.256 |
|  | Chr17:7591544 | 0.006 | 0.007 | 0.001 | 0.722 |
|  | Chr17:7591520 | 0.009 | 0.010 | 0.003 | 0.012 |
|  | Chr17:7591512 | 0.006 | 0.006 | 0.001 | 0.548 |
|  | Chr17:7591509 | 0.009 | 0.011 | 0.002 | 0.930 |
| TP53-2#1 | Chr17:7590789 | 0.027 | 0.040 | 0.018 | 0.213 |
| TP53-2#2 | Chr17:7590760 | 0.011 | 0.019 | 0.013 | 0.020 |
|  | Chr17:7590747 | 0.012 | 0.006 | -0.007 | 0.620 |
|  | Chr17:7590743 | 0.013 | 0.001 | -0.011 | 0.012 |
|  | Chr17:7590732 | 0.010 | 0.003 | -0.006 | 0.379 |
|  | Chr17:7590721 | 0.004 | 0.007 | 0.001 | 0.984 |
|  | Chr17:7590718 | 0.010 | 0.003 | -0.004 | 0.555 |
|  | Chr17:7590710 | 0.012 | 0.010 | -0.003 | 0.295 |
|  | Chr17:7590654 | 0.032 | 0.027 | -0.007 | 0.869 |
|  | Chr17:7590646 | 0.016 | 0.012 | -0.004 | 0.956 |
|  | Chr17:7590640 | 0.026 | 0.034 | 0.006 | 0.514 |
|  | Chr17:7590625 | 0.259 | 0.124 | -0.154 | 0.444 |
|  | Chr17:7590616 | 0.148 | 0.052 | -0.092 | 0.172 |
|  | Chr17:7590582 | 0.011 | 0.007 | -0.003 | 0.490 |
