# Supplementary material for: The DNA methylation of FOXO3 and TP53 as a blood biomarker of late-onset asthma
Source: J Transl Med. 2020 Dec 9;18:467. doi: 10.1186/s12967-020-02643-y (PMC7726856; doi:10.1186/s12967-020-02643-y)
Supplement: Supplementary file 3 — Additional file 3: Table S3. Correlation between mRNA expression of FOXO3 and TP53 and clinical parameters in LOA patients. [file 12967_2020_2643_MOESM3_ESM.doc]

**Table S3: Correlation between mRNA expression of FOXO3 and TP53 and clinical parameters in LOA patients.**

| **Gene** | ***p*-value** | | | | | | | |
| --- | --- | --- | --- | --- | --- | --- | --- | --- |
|  | **FEV_1_** | **FEV_1_%** | **FEV_1_/FVC** | **FVC** | **PEF** | **FEF_75_** | **FEF_50_** | **FEF_25_** |
| FOXO3 | 0.069 | 0.047* | 0.036* | 0.188 | 0.028* | 0.206 | 0.092 | 0.031* |
| TP53 | <0.000* | 0.027* | 0.779 | 0.037* | 0.029* | 0.884 | 0.506 | 0.048* |

A *p*-value < 0.05 was considered statistically significant.
