# Supplementary material for: The DNA methylation of FOXO3 and TP53 as a blood biomarker of late-onset asthma
Source: J Transl Med. 2020 Dec 9;18:467. doi: 10.1186/s12967-020-02643-y (PMC7726856; doi:10.1186/s12967-020-02643-y)
Supplement: Supplementary file 4 — Additional file 4: Table S4. Correlation between DNA methylation levels and clinical parameters in LOA patients. [file 12967_2020_2643_MOESM4_ESM.doc]

**Table S4: Correlation between DNA methylation levels and clinical parameters in LOA patients.**

| **CpG Site** | **CpG island** | ***p*-value** | | | | | | | |
| --- | --- | --- | --- | --- | --- | --- | --- | --- | --- |
|  |  | **FEV_1_** | **FEV_1_%** | **FEV_1_/FVC** | **FVC** | **PEF** | **FEF_75_** | **FEF_50_** | **FEF_25_** |
| chr6:108879441 | FOXO3-1 | 0.565 | 0.813 | 0.968 | 0.840 | 0.525 | 0.497 | 0.783 | 0.851 |
| chr6:108879922 | FOXO3-1 | 0.198 | 0.908 | 0.982 | 0.143 | 0.162 | 0.600 | 0.715 | 0.192 |
| chr6:108880271 | FOXO3-2 | 0.662 | 0.787 | 0.533 | 0.883 | 0.595 | 0.735 | 0.343 | 0.698 |
| chr6:108882982 | FOXO3-2 | 0.080 | 0.050 | 0.051 | 0.439 | 0.070 | 0.895 | 0.177 | 0.051 |
| chr6:108882977 | FOXO3-2 | 0.063 | 0.011* | 0.055 | 0.366 | 0.048* | 0.949 | 0.147 | 0.051 |
| chr6:108882964 | FOXO3-2 | 0.147 | 0.071 | 0.157 | 0.443 | 0.123 | 0.954 | 0.509 | 0.154 |
| chr6:108882825 | FOXO3-2 | 0.445 | 0.730 | 0.714 | 0.429 | 0.344 | 0.748 | 0.849 | 0.579 |
| chr17:7591672 | TP53-1 | 0.001* | 0.113 | 0.575 | <0.001* | 0.004* | 0.758 | 0.171 | 0.019* |

A *p*-value < 0.05 was considered statistically significant.
